# Epigenetic Reprogramming via Synergistic Hypomethylation and Hypoxia Enhances the Therapeutic Efficacy of Mesenchymal Stem Cell Extracellular Vesicles for Bone Repair

**DOI:** 10.3390/ijms24087564

**Published:** 2023-04-20

**Authors:** Kenny Man, Mathieu Y. Brunet, Rebecca Lees, Ben Peacock, Sophie C. Cox

**Affiliations:** 1 School of Chemical Engineering, University of Birmingham, Birmingham B15 2TT, UK; kman_888@hotmail.com (K.M.); myb925@student.bham.ac.uk (M.Y.B.); 2 NanoFCM Co., Ltd., Nottingham NG90 6BH, UK; rebecca.lees@nanofcm.com (R.L.); bpeacock@nanofcm.com (B.P.)

**Keywords:** bone, epigenetics, extracellular vesicles, tissue engineering, methylation, hypoxia, stem cells

## Abstract

Mesenchymal stem cells (MSCs) are a promising cell population for regenerative medicine applications, where paracrine signalling through the extracellular vesicles (EVs) regulates bone tissue homeostasis and development. MSCs are known to reside in low oxygen tension, which promotes osteogenic differentiation via hypoxia-inducible factor-1α activation. Epigenetic reprogramming has emerged as a promising bioengineering strategy to enhance MSC differentiation. Particularly, the process of hypomethylation may enhance osteogenesis through gene activation. Therefore, this study aimed to investigate the synergistic effects of inducing hypomethylation and hypoxia on improving the therapeutic efficacy of EVs derived from human bone marrow MSCs (hBMSCs). The effects of the hypoxia mimetic agent deferoxamine (DFO) and the DNA methyltransferase inhibitor 5-azacytidine (AZT) on hBMSC viability was assessed by quantifying the DNA content. The epigenetic functionality was evaluated by assessing histone acetylation and histone methylation. hBMSC mineralisation was determined by quantifying alkaline phosphate activity, collagen production and calcium deposition. EVs were procured from AZT, DFO or AZT/DFO-treated hBMSCs over a two-week period, with EV size and concentration defined using transmission electron microscopy, nanoflow cytometry and dynamic light scattering. The effects of AZT-EVs, DFO-EVs or AZT/DFO-EVs on the epigenetic functionality and mineralisation of hBMSCs were evaluated. Moreover, the effects of hBMSC-EVs on human umbilical cord vein endothelial cells (HUVECs) angiogenesis was assessed by quantifying pro-angiogenic cytokine release. DFO and AZT caused a time–dose dependent reduction in hBMSC viability. Pre-treatment with AZT, DFO or AZT/DFO augmented the epigenetic functionality of the MSCs through increases in histone acetylation and hypomethylation. AZT, DFO and AZT/DFO pre-treatment significantly enhanced extracellular matrix collagen production and mineralisation in hBMSCs. EVs derived from AZT/DFO-preconditioned hBMSCs (AZT/DFO-EVs) enhanced the hBMSC proliferation, histone acetylation and hypomethylation when compared to EVs derived from AZT-treated, DFO-treated and untreated hBMSCs. Importantly, AZT/DFO-EVs significantly increased osteogenic differentiation and mineralisation of a secondary hBMSC population. Furthermore, AZT/DFO-EVs enhanced the pro-angiogenic cytokine release of HUVECs. Taken together, our findings demonstrate the considerable utility of synergistically inducing hypomethylation and hypoxia to improve the therapeutic efficacy of the MSC-EVs as a cell-free approach for bone regeneration.

## 1. Introduction

Mesenchymal stem/stromal cells (MSCs) are considered a promising cell source for tissue engineering and cell therapies due to their wide availability and their multilineage potential [1,2]. Due to these favourable attributes, there have been extensive cell-based tissue engineering approaches investigated using MSCs for bone regeneration [3,4]. Despite the promising laboratory results there has been a limited translation of these MSC therapies due to issues such as functional engraftment, transplanted cell viability and uncontrolled differentiation [5,6,7]. There is a growing body of evidence demonstrating the beneficial effects of MSC-secreted bioactive factors, which are involved in paracrine signalling. One of these factors, extracellular vesicles (EVs), has been acquiring growing interest due to the intrinsic role in intercellular communication [8]. EVs are cell-derived lipid nanoparticles that contain a diverse biological cargo (i.e., proteins, nucleic acids, lipids and metabolites) involved in numerous biological processes in tissue homeostasis and development [9]. Thus, due to the translation issues of MSC-based therapeutics and the potential clinical efficacy of their EVs, the promise of MSC-EVs for bone regeneration is being widely explored.

Although there is a growing number of studies demonstrating the efficacy of MSC-EVs for bone repair [10,11], their efficacy is limited in the native state, thus hindering translation. Consequently, various bioengineering strategies have been investigated to enhance the therapeutic efficacy of MSCs and their secretome through utilising a biomimetic microenvironment [9,12,13]. It is well known that MSCs reside in hypoxic conditions, and this is involved in a number of biological processes, such as cell survival, development and differentiation [14,15]. Within a hypoxic microenvironment, the cells respond by expressing the hypoxia-inducible heterodimeric transcription factor, hypoxia-inducible factor-1α (HIF-1α), which regulates several of hypoxia-responsive genes [15,16]. During normoxia, HIF-1α is proteasomally degraded by the iron-containing prolyl hydroxylase (PHD) enzyme [17]. In hypoxic conditions, HIF-1α evades the PHD-induced degradation and translocates into the nucleus, heterodimerising with HIF-1β [18]. It has been reported that HIF-1α is an important regulator during bone repair and regeneration, mediating key biological processes such as angiogenesis and osteogenesis [19,20]. Increased skeletal vasculature and bone mass was observed in mice overexpressing HIF-1α in osteoblasts, whilst increased osteogenesis was observed in HIF-1α-transfected BMSCs [21,22]. The iron chelating agent, deferoxamine (DFO), has been demonstrated to induce cellular hypoxia by inhibiting the activity of PHD, ultimately stabilising HIF-1α expression. Moreover, it has been reported that inducing hypoxia by reduced atmospheric oxygen levels was comparable to the effects of DFO treatment [23]. Importantly, it has been shown that DFO is capable of stimulating bone formation through enhanced HIF-1α-mediated osteogenesis and angiogenesis [15,24]. Thus, DFO-induced HIF-1α expression could improve the therapeutic efficacy of MSC secretome for bone regeneration.

There has been an increasing body of evidence in the literature that the physiochemical environment in which the cells reside augments cell fate through epigenetic modifications [25,26,27]. The cells transcriptional activity is tightly controlled by post-translational mechanisms, where several studies have reported the influence of these epigenetic modifications on enhancing gene activation [28,29]. In recent years, there have been a growing number of studies demonstrating the efficacy of rewiring the epigenome of stem cells to improve their therapeutic potency for bone tissue engineering [30,31,32,33]. The potential of harnessing epigenetic reprogramming as an approach to manufacture pro-regenerative EVs has been recently demonstrated. For example, researchers exploited the impact of inducing hyperacetylation via the introduction of the histone deacetylase inhibitor (HDACi), Trichostatin A (TSA), to augment osteoblast epigenetic function, enhancing the mineralisation potential of their EVs [34]. Hyperacetylation-induced gene activation has shown great promise in regenerative medicine; however, this epigenetic modification only increases the accessibility to genes of interest, and does not directly influence their expression. Methylation is another important epigenetic mechanism that has been reported to control gene activation via augmenting chromatin remodelling and promoter activation [35]. Aberrant methylation has been reported to play a pivotal role in the silencing of tumour suppressor genes in cancer via chromatin condensation and blocking the access to promoter regions [36,37]. In the context of stem cells, pluripotency genes are usually activated when hypomethylated, while genes associated with differentiation are repressed by hypermethylation [38]. Due to the key importance of methylation in controlling stem cell functionality, there is tremendous interest in exploiting this epigenetic mechanism for biomedical applications. This has led to the development of DNA methyltransferase inhibitors (DNMTis) such as azacytidine (AZT), which incorporates into newly synthesised DNA forming covalent bonds with the DNMTs, resulting in its degradation and global genome hypomethylation [39,40]. In turn, this induced hypomethylation has been reported to promote the differentiation capacity of MSCs [41,42]; however, it is uncertain whether these pro-regenerative effects would translate into the secreted vesicles.

**Figure 1:**
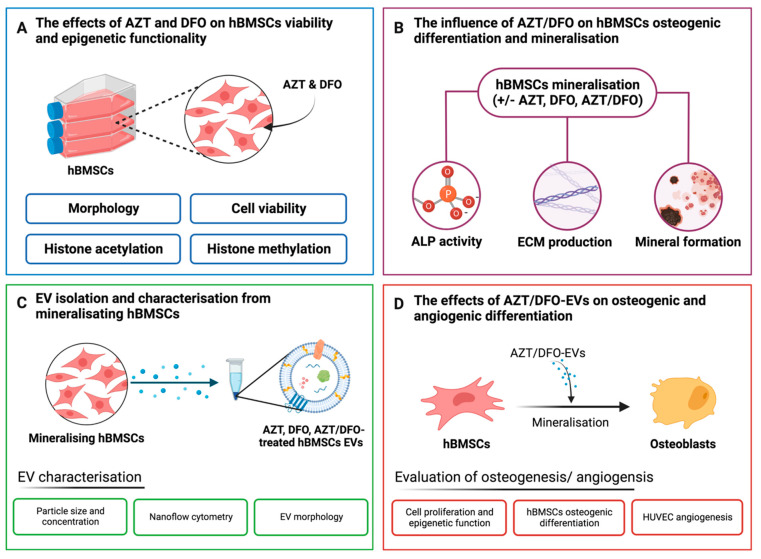
Schematic representation of the investigation of the effects of hypomethylation and hypoxia on the therapeutic potency of hBMSC-derived EVs for bone repair. (**A**) The influence of AZT and DFO on cell viability and epigenetic function was assessed. (**B**) The influence of AZT, DFO or AZT/DFO treatment on hBMSCs osteogenic differentiation evaluated by quantifying ALP activity, collagen production and calcium deposition. (**C**) EVs were isolated from untreated, AZT-treated, DFO-treated and AZT/DFO-treated hBMSCs and characterised in terms of their size, morphology and tetraspanin markers. (**D**) The effects of AZT/DFO-EVs on hBMSCs osteogenesis and HUVEC angiogenesis. Figure created with BioRender.com. (last assessed on 1 March 2023).

Interestingly, there has been growing evidence demonstrating the intrinsic relationship between epigenetic regulation and hypoxia in controlling gene expression. In normoxic conditions, the methylation of HIF-1α leads to its degradation in the nucleus independent of hydroxylation-dependant degradation [43,44]. Demethylation of HIF-1α is crucial for its stabilisation during hypoxia, enhancing its transcriptional activity. Thus, inducing hypomethylation during hypoxia could synergistically potentiate stem cell differentiation and this may in turn enhance the regenerative potency of their secreted EVs. In the present study, we investigate the synergistic influence of epigenetic reprogramming during hypoxia on enhancing the therapeutic efficacy of EVs derived from MSCs and assess their potency as an acellular tool for promoting bone regeneration. We initially determined the influence of AZT and DFO on hBMSCs epigenetic function and mineralisation capacity (Figure 1A,B). EVs were isolated from AZT-treated, DFO-treated and AZT/DFO-treated hBMSCs and characterised (Figure 1C). Isolated EVs were delivered to recipient hBMSCs and human umbilical cord vein endothelial cells (HUVECs) to evaluate their effects on osteogenic and angiogenic differentiation, respectively (Figure 1D).

## 2. Results

### 2.1. The Effects of AZT and DFO on the Morphology and Viability of hBMSCs

**Figure 2:**
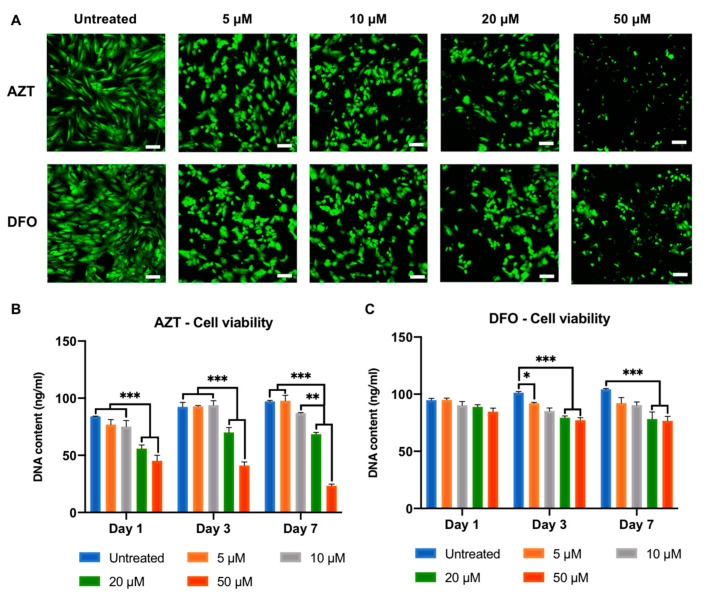
The effect of AZT and DFO on the morphology and cell viability of hBMSCs. AZT and DFO caused a time–dose dependent effect on the hBMSC (**A**) morphology and (**B**,**C**) DNA content. Data are expressed as mean ± SD (*n* = 3). * *p* ≤ 0.05, ** *p* ≤ 0.01 and *** *p* ≤ 0.001. Scale bar = 50 µm.

The effects of AZT and DFO on the viability of hBMSCs were evaluated using cellular morphology and DNA content (Figure 2). Alterations in the morphology of hBMSCs were observed upon increased dosages of both AZT and DFO, where the cells transitioned from an elongated, fibroblast-like shape to a more rounded morphology (Figure 2A). Moreover, there was a dose-dependent reduction in the quantity of live cells in both groups, indicating the time–dose dependent effects of AZT and DFO on the viability of hBMSCs. A time–dose dependent reduction in the viability of hBMSCs was observed following AZT and DFO treatment, as assessed by quantifying the DNA content (Figure 2B,C). AZT concentrations of ≥20 µM for 1 and 3 days and ≥10 µM for 7 days significantly reduced the viability of hBMSCs when compared to the untreated cells (Figure 2B). DFO concentrations of ≥5 µM for 3 days and ≥20 µM for 7 days significantly reduced the viability of hBMSCs, compared to the control group (Figure 2C).

### 2.2. AZT and DFO Pre-Treatment Promotes ALP Activity and Epigenetic Function in hBMSCs

**Figure 3:**
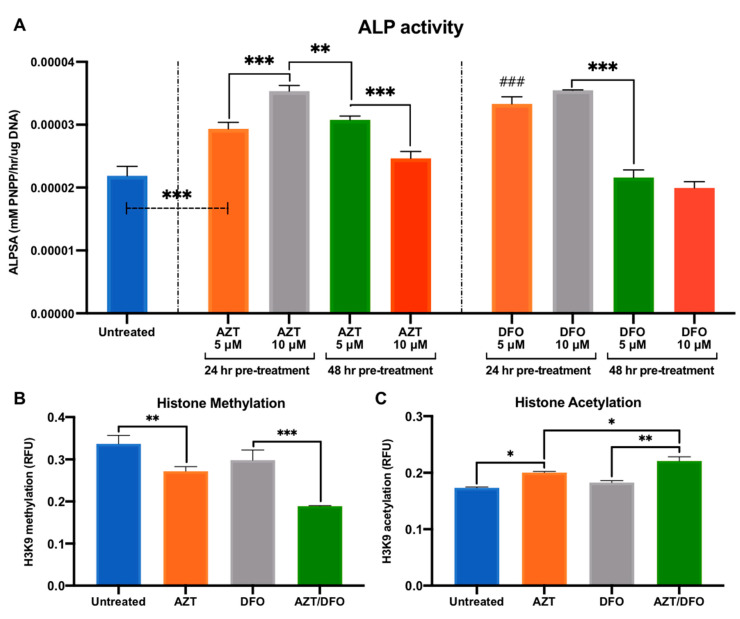
The effect of AZT/DFO pre-treatment on ALP activity and epigenetic function in hBMSCs. (**A**) ALP activity in hBMSCs pre-treated with AZT and DFO for 24 or 48 h followed by 14 days osteoinductive culture. The effect of AZT/DFO pre-treatment on hBMSC (**B**) histone methylation and (**C**) histone acetylation levels following 3 days in basal conditions. Data are expressed as mean ± SD (*n* = 3). * *p* ≤ 0.05, ** *p* ≤ 0.01 and *** *p* ≤ 0.001. ^###^ *p* ≤ 0.001 vs. untreated control.

AZT and DFO treatment resulted in time–dose dependent effects of on the viability of hBMSCs; therefore, a pre-treatment strategy was adopted to assess their effects on the osteogenesis in hBMSCs. Cells were pre-treated with AZT or DFO for 24 and 48 h, then the effects on ALP activity were assessed following 14 days osteoinductive culture (Figure 3A). An AZT pre-treatment for 24 h improved ALP activity in hBMSCs by 1.34 (5 µM) (*p* ≤ 0.001) and 1.62-fold (10 µM) (*p* ≤ 0.001), whereas a 48 h pre-treatment increased the ALP activity by 1.41 (5 µM) and 1.13-fold (10 µM), when compared to the untreated controls. DFO pre-treatment for 24 h enhanced ALP activity in hBMSCs by 1.53 (5 µM) (*p* ≤ 0.001) and 1.63-fold (10 µM) (*p* ≤ 0.001), whereas a 48 h pre-treatment caused a non-significant reduction in ALP levels by 0.99 (5 µM) and 0.91-fold (10 µM), when compared to the untreated cells. Following the time–dose dependent effect on the osteogenesis in hBMSCs, 10 µM AZT and DFO pre-treatment for 24 h was used for subsequent experiments.

The identification of these optimum treatment conditions led to an evaluation of the effects of AZT and DFO pre-treatment on the epigenetic function in hBMSCs. AZT and DFO pre-treatment significantly reduced histone methylation levels by 1.23 (*p* ≤ 0.01) and 1.14-fold when compared to the untreated control, whilst the combined AZT/DFO treatment further reduced methylation by 1.79-fold (*p* ≤ 0.001) (Figure 3B). Moreover, AZT and DFO pre-treatment increased histone acetylation by 1.16 (*p* ≤ 0.01) and 1.08-fold when compared to the untreated control, whilst combined AZT/DFO treatment further improved acetylation levels by 1.22-fold (*p* ≤ 0.001) (Figure 3C). Additionally, following immunofluorescent staining, we confirmed the enhanced expression of HIF-1α in hBMSCs following DFO treatment, when compared to the untreated control (Figure S1).

### 2.3. The Effects of Hypomethylation and Hypoxia on MSC Osteogenic Differentiation

**Figure 4:**
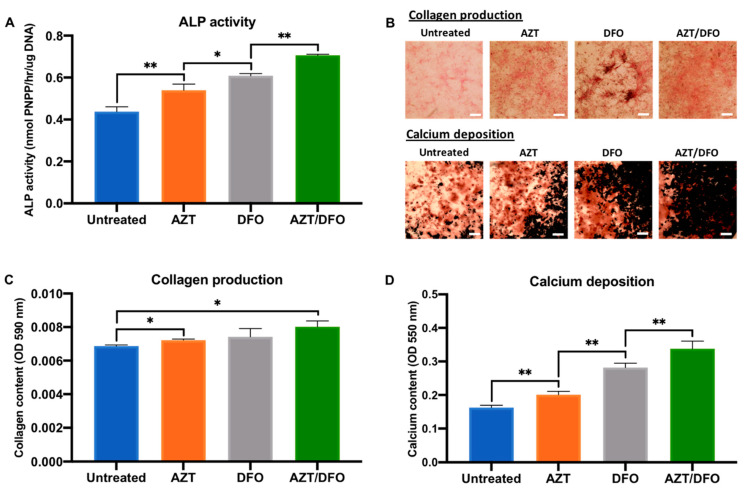
The AZT/DFO combined pre-treatment promoted osteogenic differentiation and extracellular matrix mineralisation in hBMSCs. The effects of AZT/DFO on hBMSC (**A**) ALP activity, (**B**,**C**) collagen production and (**B**,**D**) calcium deposition. Black staining indicates mineral nodule formation. Scale bar = 200 µm. Data are expressed as mean ± SD (*n* = 3). * *p* ≤ 0.05 and ** *p* ≤ 0.01.

The influence of AZT and DFO pre-treatment on osteogenic differentiation in hBMSCs was evaluated by quantifying ALP activity, collagen production and calcium deposition (Figure 4A). AZT and DFO pre-treatment significantly improved ALP activity in hBMSCs by 1.23 and 1.39-fold when compared to the untreated cells following 14 days of osteoinductive culture. AZT/DFO combined pre-treatment further improved ALP activity by 1.62-fold. Variations in ALP activity were followed by significant changes in the extracellular matrix collagen production and calcium deposition following 21 days of osteoinductive culture (Figure 4B). A quantitative analysis showed that AZT, DFO and AZT/DFO pre-treatment enhanced collagen production by 1.06, 1.09 and 1.18-fold, respectively (Figure 4C). Similarly, AZT, DFO and AZT/DFO pre-treatment increased calcium deposition by 1.23, 1.72 and 2.10-fold, respectively (Figure 4D).

### 2.4. The Isolation and Characterisation of hBMSC-Derived EVs

**Figure 5:**
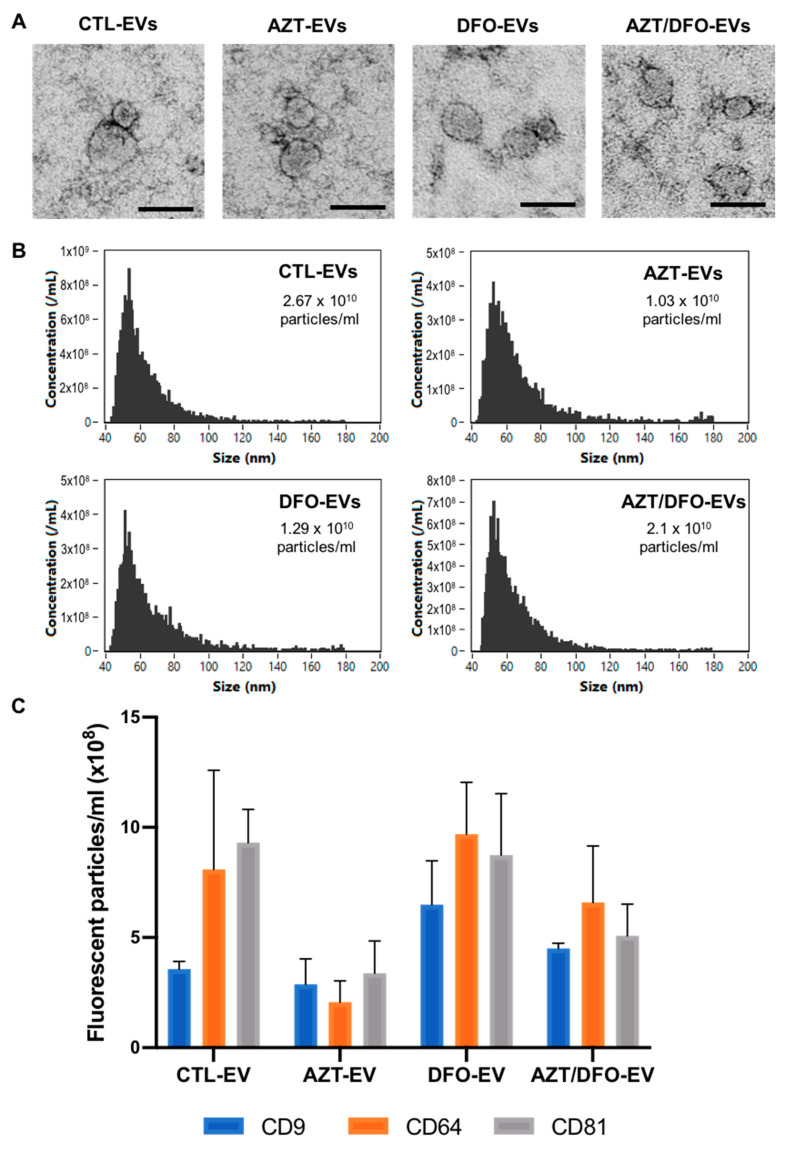
The characterisation of EVs isolated from AZT/DFO-treated hBMSCs. (**A**) TEM images of isolated EVs. Scale bar = 50 nm. (**B**) Nano-flow cytometry analysis, depicting the size distribution and concentration of particles. (**C**) Single-particle phenotyping of hBMSC-derived EVs. EVs were fluorescently labelled with APC-conjugated antibodies specific to CD9, CD63 and CD81. Data are expressed as mean ± SD (*n* = 3).

EVs from the conditioned media of untreated, AZT-treated, DFO-treated and AZT/DFO-treated hBMSCs were isolated via differential centrifugation. TEM imaging showed that EVs in all groups exhibited particles with a typical size and spherical morphology indicative of nano-sized vesicles, where they exhibit heterogeneity in their diameters (Figure 5A). The nano-flow cytometry analysis detected particles with an average diameter of 63.37 ± 17.34, 66.03 ± 20.48, 66.98 ± 21.85 and 65.33 ± 19.30 nm for the CTL-EVs, AZT-EVs, DFO-EVs and AZT/DFO-EVs, respectively (Figure 5B). A single-particle phenotyping analysis was conducted by Nanoflow cytometry. All EV groups were positive for the tetraspanin markers CD9, CD63 and CD81 (Figure 5C). Moreover, all EV groups exhibited a typical negative zeta potential (Figure S2).

### 2.5. The Effects of AZT/DFO-EVs on the General Behaviour and Epigenetic Function of hBMSCs

**Figure 6:**
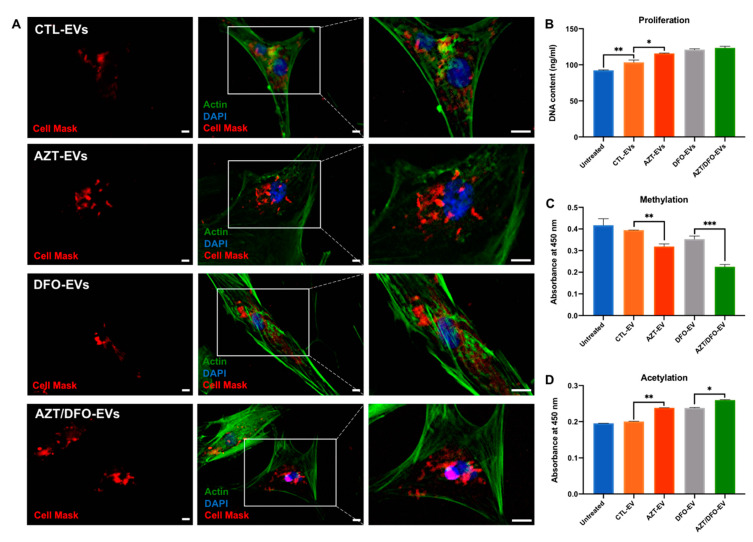
The effects of AZT/DFO-EVs on the general behaviour of hBMSCs. (**A**) EV cell uptake in hBMSCs following 16 h incubation. (**B**) Proliferation of hBMSCs treated with EVs after 7 days of basal culture. (**C**) Histone methylation levels of EV-treated hBMSCs after 3 days basal culture. (**D**) Histone acetylation levels of EV-treated hBMSCs after 3 days basal culture. Data are expressed as mean ± SD (*n* = 3). * *p* ≤ 0.05, ** *p* ≤ 0.01 and *** *p* ≤ 0.001. Scale bar = 100 µm.

The influence of MSC-EVs on the general behaviour of hBMSCs was initially evaluated. Cell Mask-labelled MSC-EVs were successfully internalised by recipient hBMSCs, where the EVs were situated within the cytoplasm of the cells (Figure 6A). Next, we evaluated the effect of these MSC-derived vesicles on hBMSC proliferation. Following 7 days of basal culture, EV-treated hBMSCs exhibited significantly increased DNA content when compared to the untreated cells (Figure 6B). Moreover, EVs derived from AZT, DFO and AZT/DFO-treated cells promoted hBMSC proliferation when compared to the CTL-EV treated group. The effects of AZT/DFO-EVs on hBMSCs epigenetic functionality was assessed by quantifying histone methylation and acetylation levels. The CTL-EV, AZT-EV, DFO-EV and AZT/DFO-EV groups reduced histone methylation levels by 1.05 (*p* > 0.05), 1.31 (*p* ≤ 0.01), 1.18 and 1.85-fold (*p* ≤ 0.001), respectively, when compared to the untreated cells (Figure 6C). The MSC histone acetylation levels were increased following EV treatment. The CTL-EV, AZT-EV, DFO-EV and AZT/DFO-EV groups increased acetylation levels by 1.03 (*p* > 0.05), 1.22 (*p* ≤ 0.01), 1.22 (*p* ≤ 0.01), and 1.33-fold (*p* ≤ 0.001), respectively.

### 2.6. AZT/DFO-EVs Enhanced the Extracellular Matrix Mineralisation in hBMSCs

**Figure 7:**
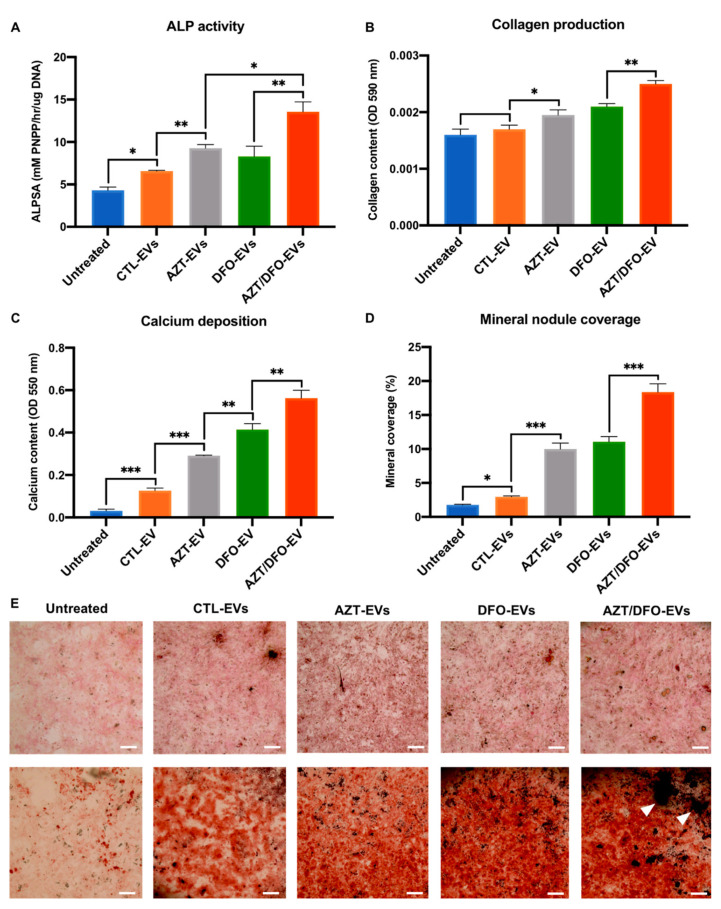
The AZT/DFO-EV treatment promoted osteogenic differentiation and extracellular matrix mineralisation in hBMSCs. The effects of AZT/DFO-EV treatment on hBMSC (**A**) ALP activity, (**B**) collagen production, (**C**) calcium deposition and (**D**) mineral nodule coverage. (**E**) Picrosirius red collagen staining and Alizarin red calcium staining of EV-treated MSCs. Black staining indicates mineral nodule formation (white arrows). Scale bar = 200 µm. Data are expressed as mean ± SD (*n* = 3). * *p* ≤ 0.05, ** *p* ≤ 0.01 and *** *p* ≤ 0.001.

The effects of AZT/DFO-EV treatment on osteogenic differentiation and mineralisation in hBMSCs were evaluated by quantifying ALP activity, collagen production, calcium deposition and mineral nodule coverage (Figure 7). ALP activity was substantially enhanced in hBMSCs treated with AZT/DFO-EVs when compared to the DFO-EV treated (1.63-fold), AZT-EVs treated (1.46-fold), CTL-EVs treated (2.06-fold) and untreated cells (3.15-fold), following 14 days in osteoinductive culture (*p* ≤ 0.05–0.01) (Figure 7A). Alterations in ALP activity in hBMSCs were followed by significant changes in extracellular matrix collagen production (Figure 7B,E). The AZT-EV (1.22-fold), DFO-EV (1.31-fold) and AZT/DFO-EV-treated (1.56-fold) groups exhibited significantly increased collagen production compared to the untreated control. Moreover, AZT-DFO-EVs treatment significantly increased extracellular matrix calcium deposition in hBMSCs when compared to the DFO-EV-treated (1.36-fold), AZT-EV-treated (1.94-fold), CTL-EV-treated (4.43-fold) and untreated cells (18.73-fold) (*p* ≤ 0.001) (Figure 7C,E). Finally, mineral nodule coverage was quantified following EV treatment. AZT-DFO-EV treatment significantly increased mineral nodule coverage (white arrows) when compared to the DFO-EV-treated (1.58-fold), AZT-EV-treated (1.84-fold), CTL-EV-treated (6.18-fold) and untreated cells (10.49-fold) (*p* ≤ 0.001) (Figure 7D,E).

### 2.7. AZT/DFO-EV Treatment Improves HUVEC Angiogenesis

**Figure 8:**
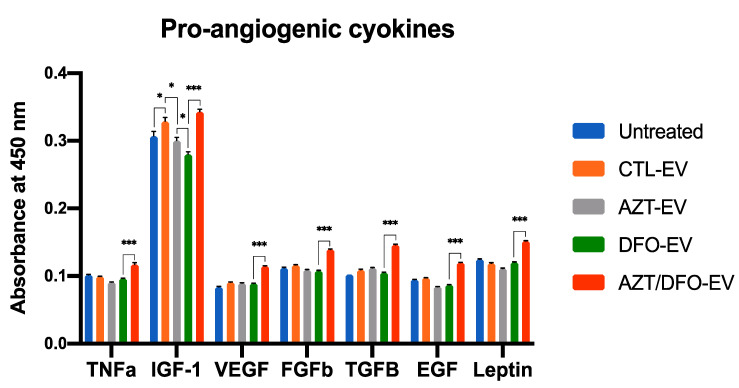
Angiogenic cytokines released from EV-treated HUVECs. The pro-angiogenic cytokine release profile of HUVECs treated with hBMSC-EVs for 3 days. Data are expressed as mean ± SD (*n* = 3). * *p* ≤ 0.05 and *** *p* ≤ 0.001.

Following the functional assessment of AZT/DFO-EVs on hBMSC osteogenesis, we evaluated the angiogenic properties of these epigenetically modified vesicles on HUVECs. Assessing the angiogenic cytokines released from EV-treated HUVECs, we showed that the AZT/DFO-EV treatment increased the production of the pro-angiogenic cytokines TNFα, IGF-1, VEGF, bFGF, TGFβ, EGF and Leptin when compared to the other EV-treated and untreated groups (Figure 8).

## 3. Discussion

**Figure 9:**
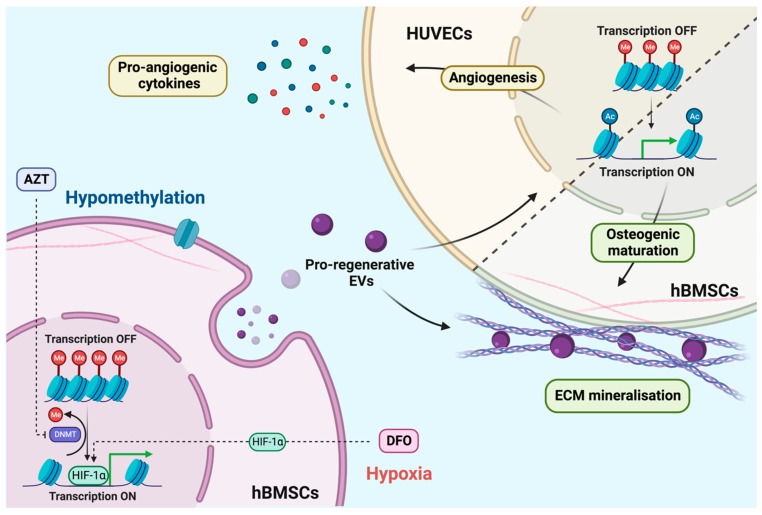
The schematic representation of how the mechanisms of AZT and DFO augment hypomethylation and hypoxia, enhancing the differentiation capacity of hBMSCs and the therapeutic potency of their EVs. Figure created with BioRender.com. (last assessed on 1 March 2023).

There has been a growing body of evidence demonstrating the potential of harnessing the regenerative capacity of EVs for numerous clinical applications [9,45]. Despite the promising utility of these natural nanoparticles, significant attention has been given to improving their clinical efficacy using a range of bioengineering approaches [9,12]. Several studies have investigated culturing cells within more biomimetic environments to improve the therapeutic potency of the EVs they secrete [46,47]. For example, MSCs are known to reside in native hypoxic conditions and are involved in a number of biological processes, such as cell survival, development and differentiation [48]. As such, it has been shown that hypoxia augments the therapeutic efficacy of MSC-EVs [49,50]. In addition to environmental conditioning, harnessing post-translational modifications (i.e., acetylation and methylation) to alter the cells epigenome has been demonstrated as an approach to control the cells transcriptional activity [4,51], ultimately enhancing the differentiation capacity and the potency of their secreted EVs [34]. Thus, augmenting the transcriptional plasticity of EV parental cells during environmental conditioning could provide a novel synergistic strategy to improve EVs’ regenerative potency for bone regeneration (Figure 9).

In the present study, we investigated the influence of altering the differentiation capacity of hBMSCs via inducing hypomethylation and hypoxia for EV production. To induce hypomethylation, we utilised the hypomethylation agent AZT that enhances the cells transcriptional activity; therefore, promoting gene expression [41,52]. To stimulate cellular hypoxia, we employed the iron-chelating agent DFO, which promotes osteogenesis through HIF-1α activation [15,24]. Following treatment with AZT and DFO, it was clear that these compounds caused a time–dose dependent alteration in the morphology of hBMSCs and significantly reduced viability (Figure 2), consistent with several studies in the literature [53]. For example, Zhou et al. showed a similar dose-dependent reduction in MSC viability following AZT treatment [41]. Whilst Mu et al. reported the time–dose dependent effect of DFO on human periodontal ligament cell viability [18]. Moreover, it has been reported that the inhibition of proliferation through cell cycle arrest, is an important transition point for the initiation of differentiation [54]. For instance, the HDACi MI192 has been shown to cause cell cycle arrest in the G_2_/M phase within human dental pulp stromal cells (hDPSCs), priming these cells with enhanced differentiation potential [28]. Thus, it is likely there is a delicate balance between the effects of AZT and DFO on hBMSC proliferation, differentiation and apoptosis.

Due to the clear influence of AZT and DFO on hBMSC viability, from an EV scalability perspective, it is critical to develop treatment approaches that do not detrimentally influence cell number or functionality for bone regeneration [55]. Several studies employing epigenetic modifications applied a pre-treatment strategy to maximise the regenerative effects without the side effects on viability [4,28]. To define the optimum treatment regimen, ALP activity during osteogenic differentiation was evaluated. ALP, is an early marker of osteoblast differentiation, playing a key role in tissue calcification and mineralisation [38]. Harnessing this approach, we demonstrated that AZT and DFO pre-treatment significantly increased ALP activity in hBMSCs (1.62-fold) when compared to the untreated cells, where cellular viability did not change over time (Figure 3). Harnessing this pre-conditioning strategy, we demonstrated the capacity of AZT and DFO to substantially promote hBMSC extracellular matrix collagen deposition and mineralisation when compared to the untreated control, following osteoinduction (Figure 4). Several studies have reported the effect of similar dosage regimens on promoting the osteogenesis of different osteoprogenitor cells [18,41]. Interestingly, simultaneous pre-treatment with AZT and DFO, further promoted the mineralisation capacity of hBMSCs when compared to the AZT or DFO-treated groups (Figure 4). Thus, it is likely the pro-regenerative effects of AZT and DFO work synergistically to promote stem cell regenerative capacity (Figure 9).

Following the confirmation that AZT and DFO pre-conditioning on hBMSCs differentiation, we investigated their effects on the cell’s epigenome. We initially evaluated the influence of AZT and DFO pre-treatment on histone methylation levels. Our results showed that the AZT/DFO treatment led to a significant reduction in histone methylation levels when compared to the AZT-treated, DFO-treated and untreated cells (Figure 3). It is expected that the DNMTi, AZT, augments the methylation status of cells; however, DFO treatment was also shown to reduce hBMSC methylation levels. It has been reported that during normoxic conditions, the transcriptional factor HIF-1α is methylated by the lysine methyltransferase SET7/9, resulting in its degradation in the nucleus [43,44]. However, in hypoxia, the demethylase LSD1 has been shown to promote the stability of HIF-1α, enhancing its transcriptional activity [56]. Thus, the reduction in methylation levels observed in DFO-treated cells indicates the successful induction of cellular hypoxia by this iron chelating agent, consistent with the enhanced HIF-1α expression observed in this study. Moreover, we evaluated the effects of pre-conditioning on histone acetylation, another key epigenetic marker of transcriptional activity. Similarly, our findings showed that the AZT/DFO-combined treatment significantly increased hBMSC histone acetylation levels to a greater degree than the AZT and DFO treatments alone (Figure 3). We did not specifically target the acetylation machinery with AZT or DFO; however, these results indicate the intrinsic link between different epigenetic mechanisms and the cells transcriptional activity, correlating with findings in the literature. Moreover, these findings demonstrate the pre-treatment strategy is capable on inducing long-term alterations in the cell epigenome, long after the removal of both AZT and DFO, consistent with the slow-binding kinetics of other epigenetic modifiers [57].

Having defined the hBMSC pre-conditioning treatment strategy, we isolated and characterised EVs derived from these cells. Our findings showed that utilising standardised procedures, we were able to isolate EVs that exhibited a typical morphology, size and protein marker expression indicative of nano-sized vesicles (Figure 5), consistent with several studies in the literature [58,59]. Moreover, it was observed that EVs isolated from the epigenetically modified cells were at a reduced quantity when compared to the CTL-EVs, a profile which correlates with the degree of osteoblast maturation, and is consistent with previous studies [34,60]. The accelerated osteogenesis induced by AZT and/or DFO treatment, enhanced the extracellular matrix production. This increased maturity in the extracellular matrix, potentially contributed to sequestering a greater quantity of released EVs in the conditioned media, due to the collagen-mediated EV capture [58,61]. Thus, the differentiation status of the parental cells potentially impacted the quantity of EVs collected.

After the confirmation of isolated EVs, we next determined the influence of these nanoparticles on the general behaviour of hBMSCs. Several studies have demonstrated the ability of these naturally derived nanoparticles to be involved in the recruitment of endogenous cells, a highly advantageous characteristic for bone augmentation strategies [62]. Our findings demonstrated that isolated EVs from all groups significantly promoted recipient hBMSC proliferation when compared to the untreated cells (Figure 6), consistent with the findings in the literature [34,60]. Interestingly, the EVs derived from the AZT-treated, DFO-treated and AZT/DFO-treated groups further promoted hBMSC proliferation when compared to the CTL-EV-treated and untreated groups. Previously, we reported that EVs derived from hyperacetylated osteoblasts significantly enhanced the recruitment of hBMSCs when compared to EVs derived from unmodified cells [34]. It has been shown that the post-translation modifications induced in the EV parental cells enriched the secreted vesicles with several microRNA species (miR-31-5p and miR-143-3p) involved in promoting cellular recruitment [34,63]. Thus, enhancing the transcriptional permissiveness of the parental hBMSCs could result in the enrichment of pro-regenerative EV cargo to promote the recruitment of recipient cells, although this would require further investigation. Critically, EVs derived from AZT, DFO and AZT/DFO-treated cells did not have a negative impact on the viability of the recipient cell population. This indicates the increased clinical utility of harnessing EVs derived from these cells when compared to employing AZT and DFO on cells directly.

There is a growing body of evidence that EVs are able to imprint the parental cell phenotype to recipient cells due to the delivery of EV-bound bioactive molecules. For instance, Shrivastava et al. demonstrated the capacity of EVs to deliver therapeutic cargo that is able to epigenetically repress Human Immunodeficiency Virus infection [64]. This inherent capability is a key reason for the increased attention on harnessing these naturally derived nanoparticles as acellular regenerative tools for different clinical applications. To determine the influence of these Epi-EVs on the recipient hBMSC transcriptional activity, we evaluated the impact on histone methylation and acetylation. Our findings showed that EVs derived from the parental hBMSCs simultaneously increased histone hypomethylation and histone acetylation levels when compared to the untreated controls (Figure 6), indicating a more permissive transcriptional machinery. The fact that CTL-EVs were able to promote the transcriptional activity of recipient cells, indicates that the EVs derived from differentiating hBMSCs were able to imprint this cellular phenotype to the EV-treated cells. This observation was confirmed with the enhanced hypomethylation and hyperacetylation observed in the AZT-EV, DFO-EV and AZT/DFO-EV-treated hBMSCs, demonstrating the successful imbuement of the altered epigenome from parental to recipient cells. This imprinting of epigenetic status to recipient cells has been observed previously, with the delivery of EVs from hyperacetylated osteoblast, resulting in histone acetylation in recipient hBMSCs [34,60]. Moreover, it was shown that EVs derived from hyperacetylated osteoblasts were enriched with microRNAs and proteins involved in transcriptional regulation, indicating a similarly altered EV cargo within the AZT/DFO-EVs, although this would require further investigation. Together, these findings suggest that modifying the hBMSCs epigenome through AZT/DFO, augments the epigenetic functionality of the recipient hBMSCs (Figure 9), mimicking the mechanism of trans-generational epigenetic inheritance reported in the literature [65,66].

Several studies have demonstrated the pro-regenerative effects of MSC-EVs for musculoskeletal conditions. For example, Jiang et al. reported the role of hBMSCs in promoting bone fracture healing in mice, due to enrichment in miR-25 [67]. Similarly, Zhai et al. showed that hBMSC-EVs were enriched with pro-angiogenic microRNAs, which targeted PI3K/Akt and MAPK signalling pathways to promote bone formation [68]. In the present study, the pro-osteogenic effects of these Epi-EVs was evaluated and our findings showed that EVs derived from AZT/DFO pre-conditioned hBMSCs significantly promoted the osteogenic differentiation and extracellular matrix mineralisation capacity of the recipient hBMSCs through the early, mid and late stages of osteogenesis following quantification of ALP activity, collagen production, calcium deposition and mineral nodule formation (Figure 7). These findings correlate with the altered epigenetic functionality following EV treatment, indicating the enhanced transcriptional permissiveness resulted in increased differentiation potential within these cells. This observation was similar to the effects of TSA-EVs on promoting the histone acetylation and mineralisation potential of hBMSCs through the delivery of pro-osteogenic microRNAs and transcriptional regulating proteins [34,60], demonstrating the considerable plasticity of harnessing epigenetic reprogramming as an approach to promote the therapeutic efficacy of EVs derived from different musculoskeletal cells. In a similar study, Diomede et al. reported the positive influence of combining hDPSCs-EVs and AZT on promoting hDPSC odontogenic differentiation within decellularized dental pulp [69]. Taken together, these findings clearly demonstrate the considerable potential of harnessing hypomethylation and hypoxia to promote the regenerative potency of hBMSCs EVs for bone regeneration (Figure 9).

Angiogenesis is an essential biological process involved in the repair of numerous tissues, particularly for bone defect repair [70,71,72]. Several studies have demonstrated the role of MSCs and their EVs in regulating blood vessel formation in the bone repair context [73,74]. Our findings showed that AZT/DFO-EVs promoted the secretion of the pro-angiogenic cytokines HUVECs when compared to the other EVs treated groups and the untreated control (Figure 8). The capacity of AZT/DFO-EVs to enhance the transcriptional activity of recipient cells, potentially improved the angiogenesis of HUVECs, resulting in the enhanced production of pro-angiogenic cytokines (Figure 9). Following the identification of the optimum AZT/DFO pre-treatment regimen to effectively enhance the regenerative potency of hBMSCs EVs in this study, the subsequent scalable manufacture of these AZT/DFO-EVs will be facilitated. This will enable future studies to investigate the regenerative capacity of these Epi-EVs within a pre-clinical critically sized bone defect model. Moreover, the scalable manufacture of these AZT/DFO-EVs will allow for an in-depth compositional analysis, providing greater understanding regarding the potential influence of the altered EV cargo (i.e., microRNAs, proteins and metabolites) on its therapeutic potency.

## 4. Materials and Methods

hBMSCs were acquired from Lonza (Lonza, Manchester, UK). The basal culture media consisted of Dulbecco’s Modified Eagle Medium (DMEM; Sigma-Aldrich, Gillingham, UK) supplemented with 10% foetal bovine serum (FBS), 1% penicillin/streptomycin (Sigma-Aldrich, Gillingham, UK) and L-glutamine (Sigma-Aldrich, Gillingham, UK). hBMSCs were used at passage 4. The mineralisation medium was comprised of basal culture media supplemented with 10 mM β-glycerophosphate (Sigma-Aldrich, Gillingham, UK), 50 μg/mL L-ascorbic acid (Sigma-Aldrich, Gillingham, UK) and 100 nM Dexamethasone (Sigma-Aldrich, Gillingham, UK). HUVECs were procured from Promega (Promega, Southampton, UK) and cultured in Endothelial Cell Growth Medium (Promega, Southampton, UK). The culture medium utilised for EV isolation and dosing was depleted of FBS-derived EVs by ultracentrifugation at 120,000× *g* for 16 h prior to use.

### 4.1. Cell Viability and Morphology Assessment

hBMSCs were seeded at 3 × 10^3^ cells/cm^2^ within a 96-well plate with a basal medium and incubated for 24 h. The medium was replaced with a fresh basal medium supplemented with/without AZT (Sigma-Aldrich, Gillingham, UK) (5, 10, 20, 50 µM) or DFO (Sigma-Aldrich, Gillingham, UK) (5, 10, 20, 50 µM), and incubated for 1, 3 and 7 days. At each time point, AlamarBlue reagent (Thermo Scientific, Paisley, UK) was added and incubated for 4 h at 37 °C. Fluorescence readings were acquired using a SPARK spectrophotometer (TECAN, Männedorf, Switzerland) at an excitation/emission wavelength of 540/590 nm, respectively. Employing the same protocol, the cell morphology was assessed via calcein-AM staining (Sigma-Aldrich, Gillingham, UK) after 3 days of culture, and observed under an EVOS fluorescent inverted microscope (Thermo Scientific, Paisley, UK).

### 4.2. Histone Acetylation and Methylation Assays

Cells were cultured in 96-well plates (3 × 10^3^ cells/cm^2^) in a basal medium for 24 h. The medium was replaced with a fresh basal medium supplemented with/without AZT (10 μM), DFO (10 μM) or AZT (10 μM)/DFO (10 μM) for 24 h, followed by 3 days of basal culture. The detection of H3K9 acetylation and methylation was performed using the EpiQuik^TM^ In Situ Histone H3-K9 Acetylation Assay Kit (Epigentek, New York, NY, USA) and EpiQuik^TM^ In Situ Histone H3-K9 Methylation Assay Kit (Epigentek, New York, NY, USA) according to the manufacturer’s protocol. The absorbance was read in a SPARK spectrophotometer at 450 nm. Histone acetylation and methylation was normalised with the DNA content.

The DNA quantification was determined by a Quant-iT PicoGreen DNA assay (Invitrogen, Life Technologies, Paisley, UK). Briefly, cells were lysed following three freeze-thaw cycles in 0.1% Triton^TM^ X-100 in phosphate-buffered saline (PBS, Lonza, Manchester, UK). A buffer of 90 μL of TE (10 mM Tris-HCl, 1 mM EDTA) was added to 10 μL of cell lysate in a 96-well plate (Corning, Deeside, UK). All samples received 100 μL of PicoGreen reagent and were incubated for 5 min. Subsequently, fluorescence was measured in a SPARK spectrophotometer at an excitation/emission wavelength of 480/520 nm.

### 4.3. HIF-1α Immunostaining

hBMSCs were seeded at a density of 4 × 10^3^ cells/cm^2^ in a chamber slide (Corning, Deeside, UK) for 24 h. Cells were treated with/without DFO (10 μM) for 24 h, then cultured in a basal medium for 3 days. Cells were washed in PBS then fixed in 10% (*v*/*v*) neutral buffered formalin (NBF, CellPath, Newtown, UK), permeabilised by 0.1% Triton^TM^ X-100 in PBS, and the non-specific binding was blocked with 10% Normal Donkey Serum (Stratech Scientific Ltd., Ely, UK) in PBS for 30 min. Samples were incubated overnight at 4 °C with the primary antibodies HIF-1α (8 μg/mL, R&D Systems, Abingdon, UK) and were washed in 0.1% Tween20 in PBS, then incubated with Donkey Anti-Mouse IgG NorthernLights (1:200, R&D Systems, Abingdon, UK). Subsequently, samples were washed in 0.1% Tween20 in PBS, stained with Alexa Fluor 488 phalloidin (1:20) (Cell Signalling Technology, Danvers, MA, USA) and mounted with Prolong^TM^ Gold Antifade Mountant with DAPI (Thermo Scientific, Paisley, UK) to label the actin cytoskeleton and nuclei, respectively. Treated cells were imaged with the EVOS fluorescent inverted microscope.

### 4.4. EV Isolation and Characterisation

#### 4.4.1. EV Isolation

hBMSCs at 80% confluence were treated with/without AZT (10 μM), DFO (10 μM), or AZT (10 μM)/DFO (10 μM) for 24 h, then cultured in a mineralisation medium for 14 days, with the medium isolated every two days. EVs were isolated from the conditioned medium by differential centrifugation: 2000× *g* for 20 min, 10,000× *g* for 30 min and 120,000× *g* for 70 min to pellet the EVs. The supernatant was removed, and the pellet was washed in sterile PBS and centrifuged at 120,000× *g* for 70 min and the resultant pellet was re-suspended in 200 μL PBS. All ultracentrifugation steps were performed utilising the Sorvall WX Ultra Series Ultracentrifuge (Thermo Scientific, Paisley, UK) and a Fiberlite, F50L-8×39 fixed angle rotor (Piramoon Technologies Inc., Santa Clara, CA, USA).

#### 4.4.2. EV Particle Size, Concentration and Tetraspanin Analysis

A NanoAnalyzer U30 instrument (NanoFCM Inc., Nottingham, UK) equipped with dual 488/640 nm lasers and single-photon counting avalanche photodiode detections (SPCM APDs) was used for simultaneous detection of side scatter (SSC) and fluorescence of individual particles. Bandpass filters allowed for the collection of light in specific channels (SSC-488/10; FL1-525/40; FL2-670/30). HPLC-grade water served as the sheath-fluid via a gravity feed, reducing the sample core stream diameter to ~1.4 µm. Measurements were taken over a 1-min interval at a sampling pressure of 1.0 kPa, maintained by an air-based pressure module. All samples were diluted to attain a particle count within the optimal range of 2000–12,000/min.

The concentration of samples was determined by comparison to 250 nm silica nanoparticles of known concentration to calibrate the sample flow rate. EV isolates were sized according to standard operating procedures using a proprietary 4-modal silica nanosphere cocktail (NanoFCM Inc., S16M-Exo). Using the NanoFCM software (NanoFCM Profession V2.0), a standard curve was generated based on the side scattering intensity of the four different silica particle populations of 68, 91, 113 and 155 nm in diameter. The laser was set to 15 mW and 10% SSC decay.

For EV tetraspanin phenotyping, the following antibodies were used: FITC-conjugated anti-human CD63 (clone MEM-259; Abcam, Cambridge, UK), FITC-conjugated anti-human CD9 (clone MEM-64; Abcam, Cambridge, UK) and FITC-conjugated anti-human CD81 (clone M38; Abcam, Cambridge, UK). An aliquot of the EV sample was diluted to 1 × 10^10^ particles/mL in PBS and 9 µL was mixed with 1 µL of conjugated antibody (single or mixed cocktail), before incubation for 30 min at room temperature. The incubation concentration ratio for single antibodies was 1:50 (1 μL of 1:5 in PBS) and 1:150 for the cocktail of 3 antibodies (1 μL of 1:5 of mixed antibody cocktail). After incubation, the mixture was diluted in PBS to 1 × 10^8^–1 × 10^9^ particles/mL for immediate phenotypic analysis.

Data processing was performed by the nFCM Professional Suite v2.0 software, with dot plots, histograms and statistical data provided in a single PDF. Gating within the software allows for proportional analysis of subpopulations separated by fluorescent intensities with the size distribution and concentration available for each sub-population.

Dynamic Light Scattering (DLS) was used to analyse the zeta potential. EV samples were diluted 1:100 in dH_2_O and the zeta potential was assessed using the Zetasizer Nano ZS (Malvern Instruments, Malvern, UK). The total EV protein concentration was determined using the Pierce BCA protein assay kit (Thermo Scientific, Paisley, UK).

#### 4.4.3. Transmission Electron Microscopy (TEM)

EVs were imaged using the JEOL JEM1400 transmission electron microscope coupled with an AMT XR80 digital acquisition system. The vesicles were physisorbed to 200-mesh carbon-coated copper formvar grids (Agar Scientific, Stansted, UK) and negatively stained with 1% uranyl acetate.

### 4.5. EV Cell Uptake

The Cell Mask^TM^ Deep Red Plasma Membrane Stain (1:1000 in PBS) (Thermo Scientific, Paisley, UK) was used to label the EVs. Following 10 min of incubation, the labelled EVs were washed twice with PBS via ultracentrifugation (120,000× *g* for 70 min). hBMSCs were seeded at a density of 4 × 10^3^ cells/cm^2^ in a chamber slide (Corning, Deeside, UK). After 24 h, the medium was replaced with a fresh basal medium supplemented with the labelled EVs. Following 16 h of incubation, the cells were fixed with 10% NBF (Cellpath, Newtown, UK), stained with Alexa Fluor 488 phalloidin (1:20) (Cell Signalling Technology, Danvers, MA, USA) and mounted with Prolong^TM^ Gold Antifade Mountant with DAPI (Thermo Scientific, Paisley, UK) to label the actin cytoskeleton and nuclei, respectively. Images were captured using the EVOS fluorescent inverted microscope.

### 4.6. EV-Induced hBMSC Osteogenesis

hBMSCs were seeded in 96-well plates (Nunc, Thermo Scientific, Paisley, UK) at a density of 21 × 10^3^ cells/cm^2^ in the basal medium. After 24 h incubation, the medium was replaced with a mineralisation medium supplemented with EVs derived from untreated (CTL-EVs), AZT-treated (AZT-EVs), DFO-treated (DFO-EVs) or AZT/DFO-treated hBMSCs (AZT/DFO-EVs) (10 µg/mL) for 21 days, unless stated otherwise. The EV-supplemented mineralisation medium changes were performed every 48 h. Cells cultured in the mineralising medium alone, without EVs, were used as the control.

### 4.7. Alkaline Phosphatase Activity

ALP activity was quantified using the 4-nitrophenyl colourimetric phosphate liquid assay (pNPP, Sigma-Aldrich, Gillingham, UK), as previously described [4]. Briefly, 10 μL of cell lysate was added to 90 μL of pNPP in a 96-well plate and incubated at 37 °C for 60 min. The absorbance at 405 nm was read on a SPARK spectrophotometer. ALP activity was then normalised with DNA content.

### 4.8. Collagen Production

Picrosirius red staining was conducted to assess extracellular matrix collagen production. Briefly, cells were washed twice in PBS, fixed in 10% NBF for 30 min, then stained with 0.1% sirius red in saturated picric acid (Sigma-Aldrich, Gillingham, UK) for 60 min. A 0.5 M acetic acid wash was used to remove the unbound dye, followed by a distilled water wash. Samples were left to air dry prior to imaging using light microscopy (EVOS XL Core, Invitrogen, Paisley, UK). To quantify collagen staining, 0.5 M sodium hydroxide was used to elute the bound dye and absorbance read at 590 nm using the SPARK spectrophotometer.

### 4.9. Calcium Deposition

Alizarin red staining was used to evaluate the extracellular matrix calcium deposition. Briefly, cells were washed twice in PBS and fixed in 10% NBF for 30 min. Samples were washed in distilled water and incubated with alizarin red solution (Sigma-Aldrich, Gillingham, UK) for 10 min. Distilled water was used to remove the unbound dye. Calcium deposition was visualised using light microscopy (EVOS XL Core, Invitrogen, Paisley, UK). For calcium quantification, bound alizarin red was eluted with 10% cetylpyridinium chloride (Sigma-Aldrich, Gillingham, UK) for 1 h and then absorbance was read at 550 nm using the SPARK spectrophotometer. The mean mineral nodule percentage coverage was quantified using ImageJ software.

### 4.10. Angiogenic Analysis

HUVECs were cultured in 96-well plates (Nunc, Thermo Scientific, Paisley, UK) at a density of 21 × 10^3^ cells/cm^2^ in a basal medium and incubated for 24 h. EVs at 10 ug/mL were added to cells and cultured for 3 days. The conditioned media was collected and the pro-angiogenic cytokines tumour necrosis factor alpha (TNFα), insulin-like growth factor 1 (IGF1), vascular endothelial growth factor (VEGF), fibroblast growth factor b (bFGF), transforming growth factor beta (TGFβ), epidermal growth factor (EGF) and leptin were all profiled using the Human Angiogenesis ELISA Strip I (Stratech Scientific Ltd., Ely, UK) according to the manufacturer’s protocol. The absorbance was read in a SPARK spectrophotometer at 450 nm.

### 4.11. Statistical Analysis

For all data presented, experiments were performed in triplicate. All statistical analysis was undertaken using ANOVA multiple comparisons test with the Tukey modification using IBM SPSS software (IBM Analytics, version 21). *P* values equal to or lower than 0.05 was considered as significant. * *p* ≤ 0.05, ** *p* ≤ 0.01 *** *p* ≤ 0.001.

## 5. Conclusions

In conclusion, these findings demonstrate that altering the epigenetic functionality of hBMSCs during hypoxia enhances the cells differentiation capacity. Importantly, the EVs derived from hypomethylated/hypoxic hBMSCs were found to significantly promote the recruitment, transcriptional activity and mineralisation potential of the recipient hBMSCs. Taken together, we have reported the considerable utility of harnessing synergistic epigenetic reprogramming during hypoxia to improve the manufacture of pro-regenerative EVs as a multifunctional nanoscale therapeutic tool to promote bone formation.

## Data Availability

The data presented in this study are available on request from the corresponding author.

## Supplementary Materials

The following supporting information can be downloaded at: https://www.mdpi.com/article/10.3390/ijms24087564/s1.
