# Supplementary material for: Epigenetic Reprogramming via Synergistic Hypomethylation and Hypoxia Enhances the Therapeutic Efficacy of Mesenchymal Stem Cell Extracellular Vesicles for Bone Repair
Source: Int J Mol Sci. 2023 Apr 20;24(8):7564. doi: 10.3390/ijms24087564 (PMC10142722; doi:10.3390/ijms24087564)
Supplement: Supplementary file 1 [file ijms-24-07564-s001.zip › ijms-2324507-supplementary.pdf]

Supplementary materials

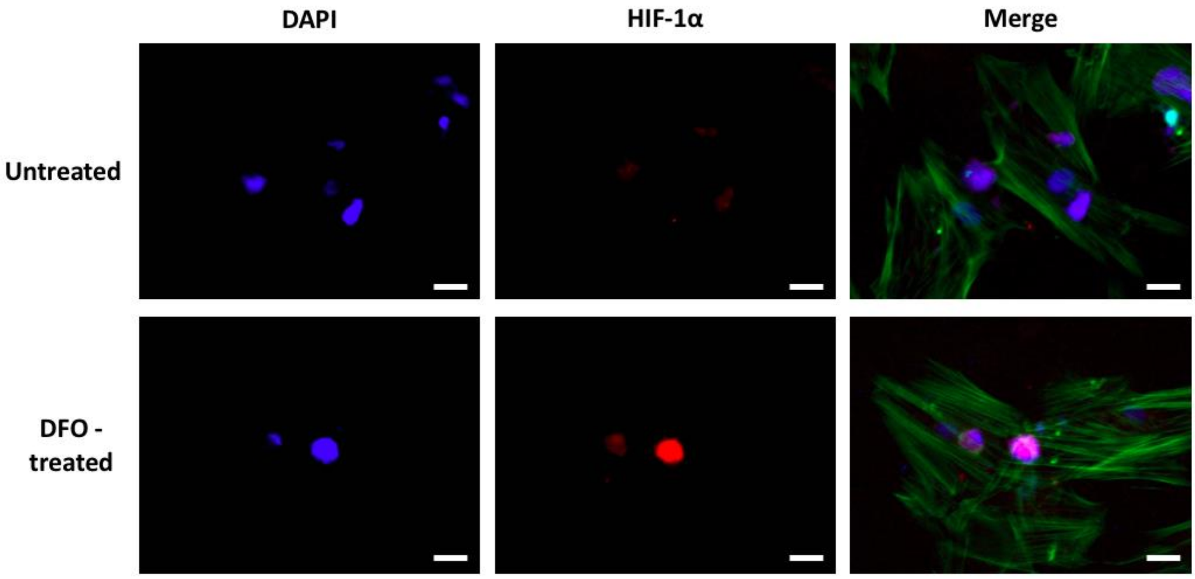

Figure S1. Immunofluorescent staining of HIF-1α in untreated and DFO-treated hBMSCs. Scale bar = 100 μm.

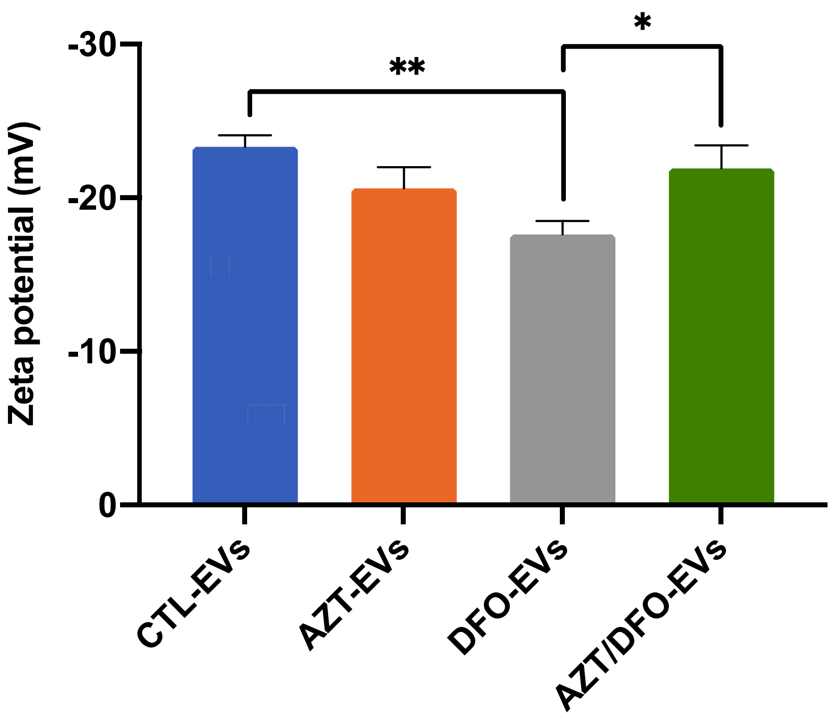

Figure S2. Zeta potential of hBMSCs-derived EVs. Data are expressed as mean ± SD (n=3). *$P \leq 0.05$ and **$P \leq 0.01$.
